# TSPO safeguards porphyrin–iron balance under anoxic conditions in *Bacillus cereus*

**DOI:** 10.1128/msystems.01738-25

**Published:** 2026-04-13

**Authors:** Catherine Duport, Caroline Schmitt, Jean Armengaud

**Affiliations:** 1 Avignon Université, INRAE, UMR SQPOV 26998 , Avignon, France; 2 Assistance Publique Hôpitaux de Paris (AP-HP), Hôpital Louis Mourier, Centre Français des Porphyries, Colombes, France; 3 Université Paris Cité, INSERM, EFS, BIGR, Paris, France; 4 Département Médicaments et Technologies pour la Santé (DMTS), Université Paris Saclay, CEA, INRAE, SPI, Bagnols-sur-Cèze, France; National Institutes of Health National Institute of Allergy and Infectious Diseases, Bethesda, Maryland, USA

**Keywords:** TSPO, tetrapyrrole metabolism, iron homeostasis, redox imbalance, anoxic fermentation, *Bacillus cereus*, proteomics

## Abstract

The physiological role of the bacterial translocator protein (TSPO) under anoxic conditions remains poorly defined. Here, we show that deletion of *tspO* in *Bacillus cereus* growing under fermentative anoxic conditions leads to increased porphyrin accumulation, redox imbalance, and impaired growth. Cellular proteome analyses reveal extensive remodeling of central metabolism, including increased abundance of proteins in the siroheme branch of tetrapyrrole biosynthesis, increased abundance of nitrate assimilation and respiratory nitrate reduction components, and broad alterations in iron, metal ion, and phosphate homeostasis. The Δ*tspO* mutant also displays a characteristic nitrosative stress signature, with sustained induction of NO-detoxifying systems. These combined defects result in marked changes in the exoproteome. Taken together, our findings support a model in which *B. cereus* TSPO links porphyrin metabolism to intracellular iron utilization, thereby supporting heme biosynthesis and redox balance under anoxic conditions.

**IMPORTANCE:**

Understanding how bacteria maintain metabolic and redox balance under anoxic conditions is essential for deciphering how they adapt to fluctuating environments. Here, we identify the translocator protein *Bacillus cereus* TSPO (BcTSPO) as a key coordinator of porphyrin and iron metabolism in *Bacillus cereus*, contributing to the proper functioning of the heme biosynthetic pathway and limiting the accumulation of toxic intermediates. Loss of BcTSPO triggers redox and nitrosative stress, ion dysregulation, and broad metabolic instability, highlighting its central role in maintaining cellular homeostasis during oxygen deprivation. These findings extend the physiological scope of bacterial TSPO beyond oxygen-dependent contexts and support the idea that its ancestral function lies in coordinating tetrapyrrole metabolism with intracellular iron use to sustain cellular resilience across diverse environmental conditions.

## INTRODUCTION

The translocator protein (TSPO) is an evolutionarily conserved protein found in mitochondria and other cellular membranes across eukaryotes, and in the plasma membrane of bacteria (1, 2). In mammals, TSPO contributes to mitochondrial bioenergetics, steroidogenesis, and immune responses, and its upregulation during injury or disease underscores a broader role in cellular stress adaptation (3). In plants, TSPO contributes to abiotic stress adaptation via abscisic acid-dependent signaling pathways (4, 5). In bacteria, it has been linked to diverse adaptive processes. In *Rhodobacter sphaeroides*, TSPO regulates porphyrin export and photosynthetic gene expression in response to oxygen availability (6). Similar roles have been proposed in *Dinoroseobacter shibae* and *Fremyella diplosiphon* (7, 8), while in *Pseudomonas Pf0-1* and *Sinorhizobium meliloti*, TSPO contributes to osmotic and nutrient stress responses, respectively (9, 10). Across biological kingdoms, TSPO has been repeatedly linked to redox regulation, although its precise role—whether protective or signaling—remains debated (2, 3). Despite this broad conservation, the molecular mechanisms underlying TSPO function remain poorly defined. Functional complementation across domains of life, such as mammalian TSPO restoring photosynthetic regulation in a TSPO-deficient *Rhodobacter sphaeroides* mutant (6), or a chimeric mouse–*Arabidopsis* TSPO reproducing the plant protein’s function (11), supports the existence of a conserved yet adaptable mechanism linking TSPO to cellular homeostasis.

*Bacillus cereus* is a facultative anaerobic pathogen (12) that colonizes oxygen-limited environments, including the intestinal lumen, where it relies on fermentation or anaerobic respiration using nitrate or nitrite as terminal electron acceptors (13). This metabolic flexibility makes *B. cereus* a suitable model to investigate cellular mechanisms that maintain redox and metabolic homeostasis across fluctuating oxygen conditions. In particular, porphyrin metabolism lies at the intersection of respiratory processes and redox balance, and therefore provides a relevant framework to explore the physiological role of TSPO in bacterial stress adaptation. Previous work showed that *B. cereus* TSPO (BcTSPO) prevents porphyrin accumulation and oxidative damage under oxic conditions (14), and can degrade protoporphyrin IX *in vitro* (15, 16). These results suggest that BcTSPO contributes to porphyrin turnover, redox protection, and potentially virulence regulation (17). Given its established role in porphyrin metabolism and oxidative stress protection under oxic conditions, we hypothesized that BcTSPO may contribute to intracellular homeostasis independently of oxygen availability. Specifically, we asked whether BcTSPO participates in redox and metabolic regulation during fermentative anoxia, where oxidative stress is minimal but metabolic adaptation remains essential. To test this hypothesis, we performed comprehensive comparative analyses of the ∆*tspo* mutant and the ATCC 14,579 wild-type (WT) strain under fermentative anoxic conditions. Using growth assays, metabolic measurements, and quantitative cellular and exoproteomic profiling, we assessed the impact of BcTSPO absence on cellular physiology. Our analyses revealed that BcTSPO contributes to intracellular homeostasis under oxygen deprivation and uncovered an unexpected link between BcTSPO, porphyrin–iron metabolism, and redox balance. These findings extend the functional scope of TSPO beyond oxidative stress protection and identify BcTSPO as a key coordinator of metabolic stability under anoxic conditions.

## RESULTS

### BcTSPO modulates anaerobic glucose fermentation

Under fermentative anoxic conditions in MODG medium, the Δ*tspO* strain showed a reduced maximal specific growth rate (µ_max_) and final biomass yield compared to WT (Table 1). This phenotype was accompanied by lower glucose consumption, reflecting a decrease in overall metabolic flux. Notably, a larger proportion of metabolized glucose was redirected toward acetate production in the Δ*tspO* mutant, whereas the yields of other fermentation by-products remained unchanged. Despite this shift in carbon output, the biomass yield per mole of ATP was similar between the Δ*tspO* mutant and WT, indicating that the energetic efficiency of anaerobic growth was not compromised. Together, these results show that BcTSPO modulates carbon-flux partitioning and supports optimal fermentative growth under anoxic conditions.

**TABLE 1:** Glucose fermentation parameters of wild-type (WT) and Δ*tspO* mutant strains grown in controlled anaerobic batch cultures (pH 7.2, pO₂ = 0%) in MODG medium^*a*^

Parameter	WT	Δ*tspO*
Growth parameters		
Final biomass (g·L^−1^)	1.84 ± 0.09*	1.23 ± 0.04*
µ_max_ (h^−1^)	0.96 ± 0.05*	0.64 ± 0.14*
Glucose consumption (%)	72 ± 13*	45 ± 7*
Y biomass/glucose (g·g^−1^)	2.01 ± 0.27	1.86 ± 0.23
Y biomass/ATP (g·mol^−1^)	45.40 ± 6.19	48.75 ± 5.89
Yields of end products (mol/mol of glucose)		
Lactate	1.34 ± 0.12	1.44 ± 0.15
Acetate	0.19 ± 0.02*	0.28 ± 0.05*
Formate	0.42 ± 0.02	0.41 ± 0.09
Ethanol	0.15 ± 0.06	0.12 ± 0.05
Succinate	NZ	NZ
ATP	2.00 ± 0.00	2.00 ± 0.00
Pyridine nucleotides		
NAD^+^	4.23 ± 0.06*	3.24 ± 0.11*
NADH	0.90 ± 0.06*	0.34 ± 0.04*
NADP^+^	0.87 ± 0.23*	1.46 ± 0.34*
NADPH	8.22 ± 0.87*	0.87 ± 0.34*
NADPH/NADH	9.12 ± 0.94*	2.37 ± 1.20*
NADP^+^/NAD^+^	0.20 ± 0.10*	0.45 ± 0.10*

^*a*^ Values represent means ± standard deviation (SD) from three independent biological replicates. Asterisks indicate statistically significant differences between strains (Student’s *t*-test, *P* < 0.05). NZ, near zero.

### BcTSPO limits porphyrin accumulation under anaerobiosis

**Fig 1:**
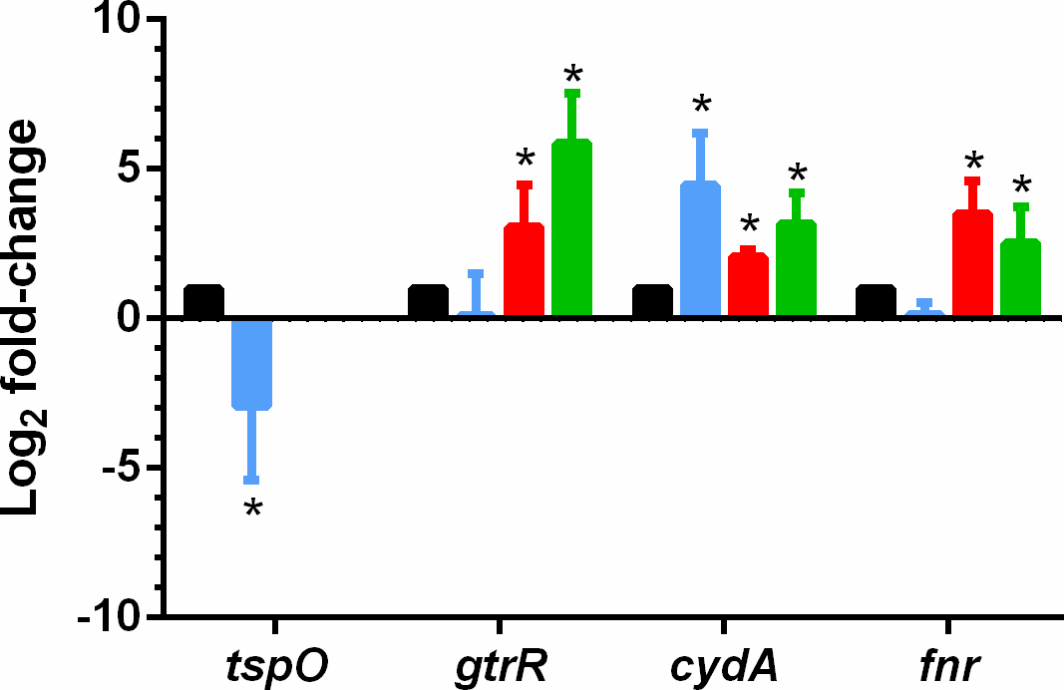
Differential expression of *tspO* and associated genes. Samples from WT and Δ*tspO* strains were collected at exponential and stationary growth phases. Transcript levels of *tspO*, *gntR*, *cydA*, and *fnr* were quantified by RT-qPCR, normalized to the 16S rRNA gene (*ssu*), and expressed as fold changes relative to the WT strain at the exponential phase (set to 1, black). Log₂ fold change values are shown for the WT at stationary phase (blue) and for the Δ*tspO* mutant at exponential (red) and stationary (green) phases. Data represent the mean ± SD of six measurements (three biological replicates with two technical replicates each). Statistical significance was assessed using Student’s *t*-test; *, *P* < 0.05. *tspO* transcripts were undetectable in the Δ*tspO* mutant.

**Fig 2:**
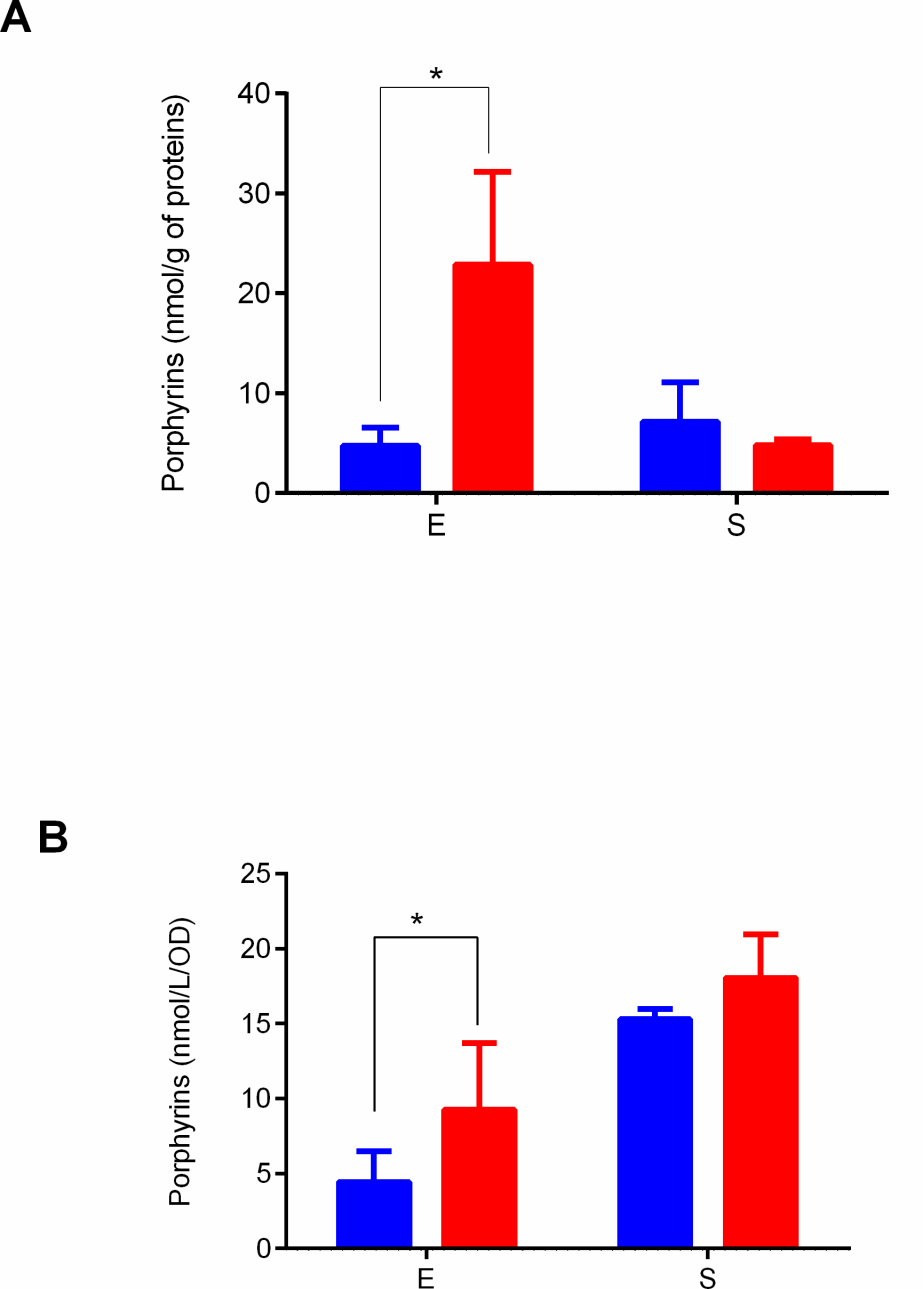
Intracellular accumulation and extracellular excretion of total porphyrins in WT (blue) and Δ*tspO* (red) cells at exponential (E) and stationary (S) growth phases. (**A**) Intracellular porphyrin content measured in cell pellets. (**B**) Extracellular porphyrin content measured in culture supernatants. Results are presented as mean ± SD from three biological replicates. Statistical significance was assessed using Student’s *t*-test; *, *P* < 0.05.

Given that BcTSPO modulates carbon flux under fermentative anoxia and has been previously implicated in porphyrin turnover and redox regulation (14), we quantified intracellular and extracellular porphyrin levels during exponential (E) and stationary (S) growth phases (Fig. 2; Fig. S1). These time points were selected because *tspO* peaks during exponential growth (Fig. 1). The results show that both intracellular and extracellular porphyrin levels were significantly higher in the ∆*tspO* mutant compared to WT at the E phase, whereas no significant difference was observed at the S phase (Fig. 2). The predominant porphyrins identified in the extracellular medium, averaged across the two strains and the two growth phases tested, were coproporphyrin (85% ± 4%) and uroporphyrin (10% ± 3%), which together accounted for more than 95% of the porphyrins detected (data not shown). Intracellular porphyrin speciation was more heterogeneous than in the extracellular medium, with coproporphyrin remaining predominant (56% ± 10%), followed by pentacarboxylporphyrin (28% ± 13%), while other species were present at lower proportions (uroporphyrin 9% ± 6%, heptacarboxylporphyrin 4% ± 2%, hexacarboxylporphyrin 3% ± 1%). Interestingly, extracellular porphyrin concentrations under anoxia were more than 10-fold higher than those previously measured under oxic conditions (14), indicating that fermentative metabolism strongly enhances porphyrin release. Together, these data show that BcTSPO restrains porphyrin accumulation during active anaerobic fermentative growth.

To confirm that the growth and porphyrin phenotypes observed under fermentative anoxic conditions were directly attributable to *tspO* deletion, we performed plasmid-based complementation assays. As shown in Fig. S1, expression of *tspO* in the Δ*tspO* background restored growth parameters and significantly reduced intracellular porphyrin levels toward wild-type values.

### BcTSPO shapes both the cellular proteome and the exoproteome

**Fig 3:**
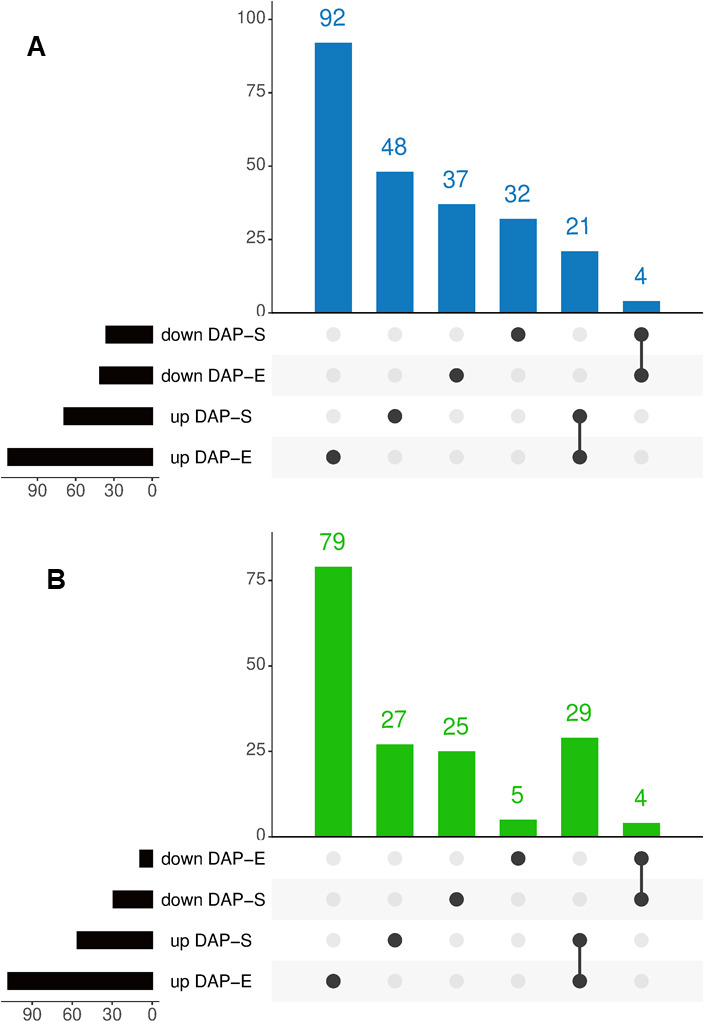
Intersection of differentially abundant proteins (DAPs) between exponential (E) and stationary (S) phases in the Δ*tspO* mutant compared to wild type (WT). (**A**) UpSet plot illustrating the number of shared and specific up- and down-DAPs in the cellular proteome at E and S phases. (**B**) UpSet plot showing the overlap of up- and down-DAPs in the exoproteome at E and S phases. Proteins were considered differentially accumulated with a |log₂ fold change| > 0.58 and a *P*-value <0.05.

To assess the global impact of BcTSPO on *B. cereus* physiology, we compared the cellular and extracellular proteomes of the Δ*tspO* mutant and WT strains at the E and S phases (Fig. S1). Across all samples, 1,803 cellular proteins and 935 extracellular proteins were confidently identified (Tables S1 and S2). In the cellular proteome, 234 proteins were differentially abundant in the Δ*tspO* mutant (|log₂ fold change| > 0.58, *P* < 0.05): 113 increased and 41 decreased at the E phase, and 69 increased and 36 decreased at the S phase (Fig. 3A). Twenty-one increased and four decreased proteins were shared between phases, indicating a persistent intracellular signature associated with *tspO* deletion. In the exoproteome, 169 proteins were differentially abundant: 108 increased and 9 decreased at E phase, and 56 increased and 29 decreased at S phase (Fig. 3B). Thirty-three of these were shared across phases, forming a stable extracellular signature of the Δ*tspO* phenotype. These results indicate that the absence of BcTSPO causes broad and dynamic remodeling of both the cellular proteome and exoproteome.

### BcTSPO modulates core metabolic functions and stress-associated pathways

**Fig 4:**
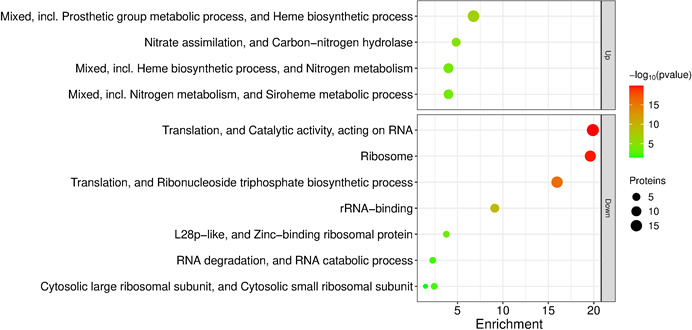
Bubble plot of cluster enrichment analysis of up- and down-differentially abundant proteins (DAPs) identified in the cellular proteome during the exponential growth phase. Clusters corresponding to upregulated DAPs are displayed in the upper panel, while clusters corresponding to downregulated DAPs are shown in the lower panel. The *x*-axis indicates the proportion of proteins associated with each functional category (cluster), while the bubble size reflects the number of DAPs within each cluster. Only statistically significant enrichments (STRING, *P* ≤ 0.05) are shown.

To identify the functional consequences of *tspO* deletion, we performed cluster enrichment analysis of cellular differentially abundant proteins (DAPs). At the E phase, the Δ*tspO* mutant displayed a marked decrease in ribosomal and translation-associated proteins, together with an increased abundance of proteins involved in tetrapyrrole metabolism and nitrogen metabolism (Fig. 4). In contrast, no significant functional enrichment was detected at the S phase, consistent with the more limited and heterogeneous cellular proteomic changes observed at this stage. These results indicate that the absence of BcTSPO triggers a pronounced but transient metabolic reprogramming during active growth, likely reflecting the cellular response to porphyrin accumulation and redox stress, which subsides once cultures enter the stationary phase (Fig. 2).

### BcTSPO modulates tetrapyrrole and nitrogen metabolism

**Fig 5:**
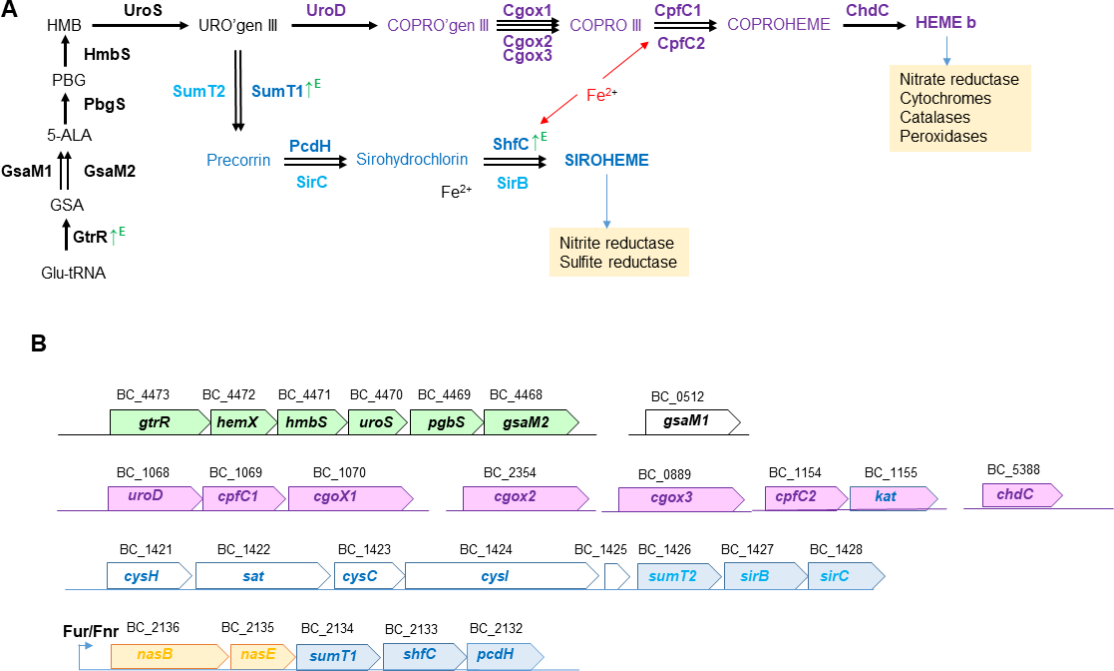
Tetrapyrrole biosynthesis pathways and genomic organization of related genes in *Bacillus cereus*. (**A**) Schematic representation of the heme and siroheme biosynthetic pathways highlighting enzymes identified in the proteomic analysis. ↑^E^ denotes proteins with significantly increased abundance during the exponential growth phase in the Δ*tspO* mutant relative to the wild type. (**B**) The inset shows the genomic organization of gene clusters encoding enzymes involved in heme and siroheme biosynthesis. GtrR, glutamyl-tRNA reductase; GsaM1/GsaM2, glutamate-1-semialdehyde aminotransferases; PbgS, porphobilinogen deaminase; HmbS, hydroxymethylbilane synthase; UroS, uroporphyrinogen-III synthase; UroD, uroporphyrinogen decarboxylase; CgoX1/CgoX2/CgoX3, coproporphyrinogen III oxidases; CpfC1/CpfC2, coproporphyrin III ferrochelatases; ChdC, coproheme decarboxylase; SumT, uroporphyrinogen-III C-methyltransferase; PcdH/SirC, precorrin-2 dehydrogenases; ShfC/SirB, CbiX proteins; KatA, catalase; CysH, phosphoadenylyl-sulfate reductase; Sat, dissimilatory-type sulfate adenylyltransferase; CysC, adenylylsulfate kinase; CysI, ferredoxin–sulfite reductase; NasB, nitrite reductase large subunit; NasE, nitrite reductase small subunit.

In line with the enrichment of tetrapyrrole and nitrogen metabolism pathways in the Δ*tspO* mutant (Fig. 4) and the altered porphyrin levels observed (Fig. 2), we examined in detail the proteins involved in tetrapyrrole and nitrogen metabolism. Cellular proteomic data showed that glutamyl-tRNA reductase (GtrR), the first committed enzyme of the heme biosynthesis pathway, was more abundant in the Δ*tspO* mutant at the E phase (Fig. 5A; Table S1), consistent with the transcriptional activation of *gtrR* (Fig. 1). In contrast, enzymes mediating the downstream conversion of glutamate-1-semialdehyde to uroporphyrinogen III—particularly those encoded in the *gntR*-associated operon (Fig. 5B)—remained unchanged, suggesting a metabolic bottleneck at this step. Uroporphyrinogen III feeds into both heme and siroheme biosynthesis. No significant changes were detected in the abundance of enzymes in the heme branch, whereas two siroheme-specific enzymes, the SAM-dependent uroporphyrinogen III methyltransferase (SumT1) and sirohydrochlorin ferrochelatase (SfhC), were more abundant in the Δ*tspO* mutant at the E phase (Fig. 5A). These enzymes are encoded within the same genomic locus as *nasB* and *nasE*, which encode the large and small subunits of the assimilatory nitrite reductase. NasB and NasE proteins also increased in abundance in the Δ*tspO* mutant compared to WT (Table S1). Analysis of the upstream regulatory region of *nasB* revealed predicted Fnr and Fur binding sites (Fig. 5B). Fur abundance decreased in the Δ*tspO* mutant (Table S1), while *fnr* transcript levels increased at both E and S phases (Fig. 1), indicating transcriptional activation of the *nas* operon. Within the siroheme pathway (Fig. 5A), enzymes dedicated to the sulfite reduction branch (SumT2, SirB, SirC) were absent or detected only at low levels, indicating preferential activation of nitrate assimilation over sulfite reduction.

**Fig 6:**
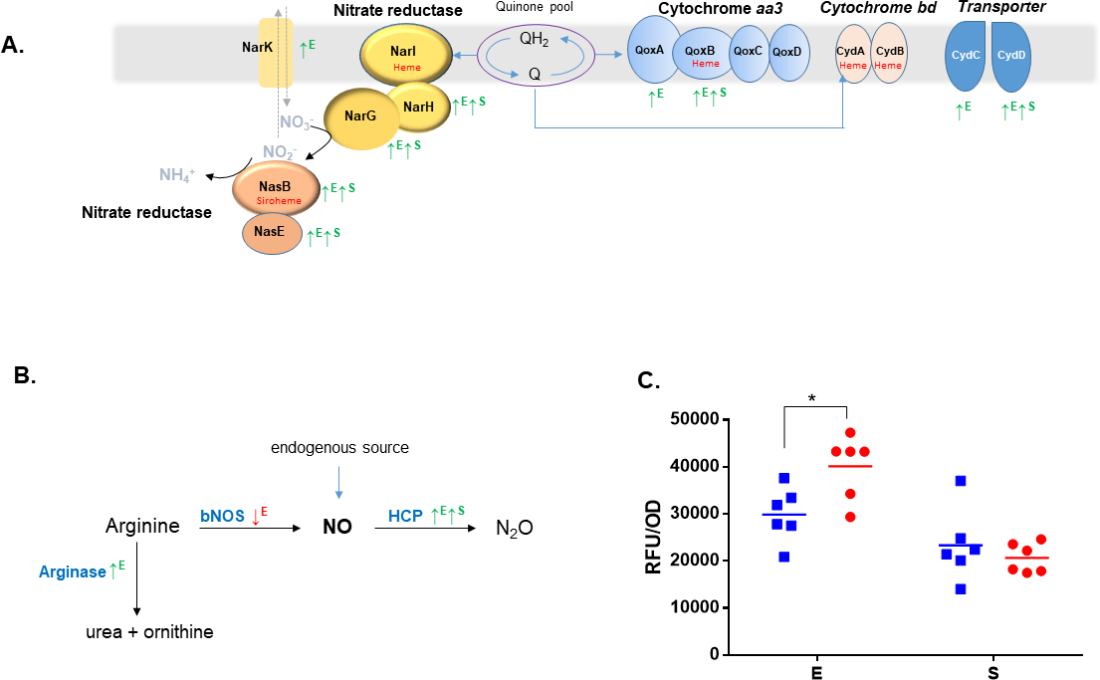
Altered respiratory components and nitrosative stress markers in the Δ*tspO* mutant. (**A**) Schematic representation of key nitrogen metabolism and respiratory chain components detected in the proteomic analysis, including NADH-dependent nitrite reductase (NasBE), membrane-bound nitrate reductase complex (NarGHI), nitrate/nitrite antiporter (NarK), terminal oxidases (cytochromes *aa₃* and *bd*), and CydCD transporter. Although nitrate (NO₃⁻), nitrite (NO₂⁻), and ammonium (NH₄^+^) are absent from the growth medium, their corresponding metabolites are shown in gray to reflect their biological relevance and the potential activity of the associated enzymes. ↑^E^ denotes proteins with significantly increased abundance during the exponential growth phase in the Δ*tspO* mutant relative to the wild type. ↑S indicates increased abundance during the stationary phase. (**B**) Schematic overview of endogenous nitric oxide (NO) metabolism pathways. The diagram includes NO production from arginine via nitric oxide synthase (NOS), detoxification via hybrid cluster protein (Hcp), and alternative arginine catabolism via arginase. ↑^E^ denotes proteins with significantly increased abundance during the exponential growth phase in the Δ*tspO* mutant relative to the wild type. ↑S indicates increased abundance during the stationary phase; ↓E indicates decreased abundance during the exponential growth phase. (**C**) Intracellular ROS/RNS levels measured with the general redox-sensitive probe DCFDA (expressed as RFU) in WT (blue) and Δ*tspO* mutant (red) cells. * indicates *P* < 0.05 according to Student’s *t*-test.

Surprisingly, several components of the respiratory nitrate reduction pathway were also increased in the Δ*tspO* mutant, despite the absence of nitrate or nitrite in the medium. These included the catalytic and electron-transfer subunits of the NarGHI complex (NarG, NarH), the nitrate/nitrite transporter NarK (Fig. 6A), and enzymes involved in molybdenum cofactor biosynthesis (MoaA, MoeA, MoeB). Proteins associated with aerobic respiration were likewise increased: the *aa₃*-type cytochrome oxidase complex (QoxA, QoxB) accumulated during both the E and S phases, and *cydAB* transcripts were elevated (Fig. 1), although the corresponding proteins remained undetected (Table S1). Taken together, these findings suggest remodeling of interconnected tetrapyrrole-dependent, respiratory, and nitrogen metabolic pathways in the Δ*tspO* mutant under fermentative anoxic conditions.

### BcTSPO modulates nitric oxide homeostasis and redox balance

Several proteomic markers pointed to a disturbance in nitric oxide (NO) homeostasis in the Δ*tspO* mutant (Table S1). At the E phase, nitric oxide synthase (bNOS) was less abundant in Δ*tspO* cells, whereas the hybrid cluster protein (Hcp), involved in nitric oxide detoxification (18), was more abundant. The increased abundance of Hcp persisted at the S phase, suggesting a sustained engagement of NO detoxification mechanisms (Fig. 6B; Table S1). Additional evidence for altered NO homeostasis included increased abundance of NasD and NasE, components of the assimilatory nitrite reductase complex known to be induced by NO, and of the CydDC transporter (Fig. 6A), which contributes to cytochrome *bd* assembly and resistance to nitrosative stress (19, 20). Consistently, intracellular levels of reactive oxygen and nitrogen species (ROS/RNS) measured using the general redox-sensitive probe 2′,7′-dichlorofluorescein diacetate (DCFDA) were significantly higher in Δ*tspO* cells compared to the WT strain (Fig. 6C), supporting the occurrence of nitrosative stress under fermentative anoxic conditions.

**Fig 7:**
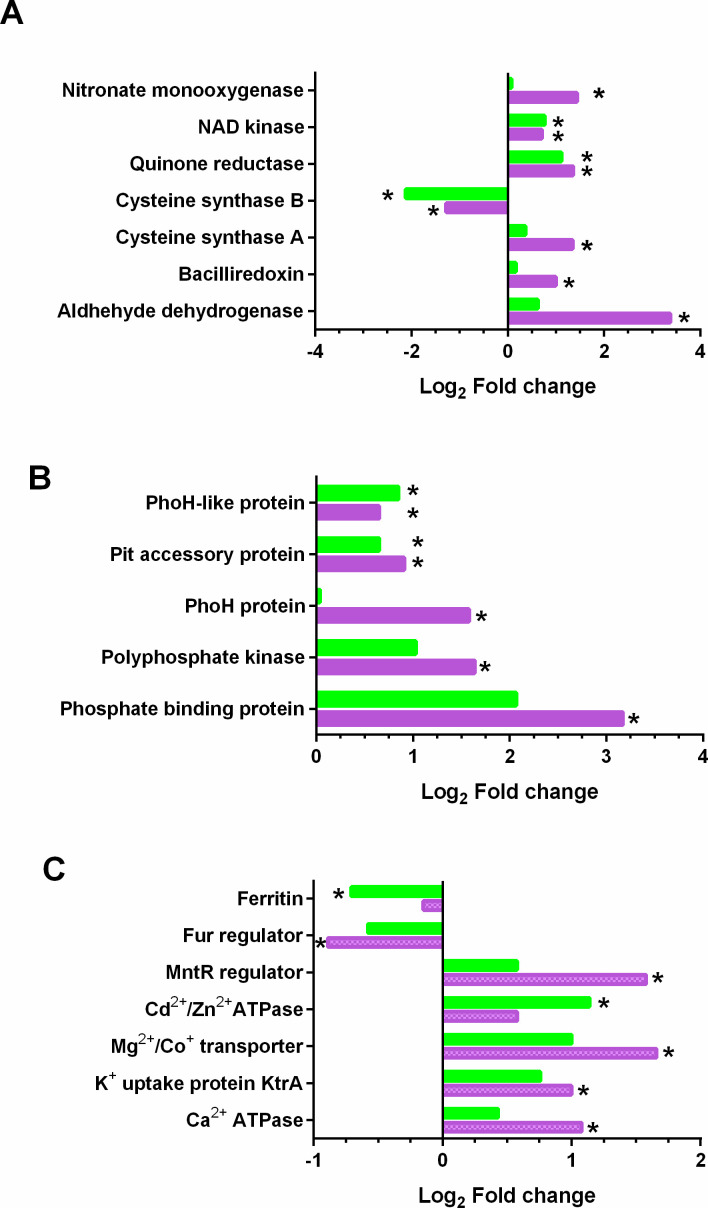
Alterations in redox cofactor metabolism and stress-related pathways in Δ*tspO* cells. (**A**) Proteins involved in redox cofactor metabolism showing differential abundance in the Δ*tspO* mutant compared to the WT strain. (**B**) Differentially abundant proteins involved in phosphate metabolism. (**C**) Differentially abundant proteins related to ion homeostasis. Bars represent log₂ fold change (FC) in protein abundance relative to WT (positive values indicate increased abundance, negative values indicate decreased abundance) during exponential (pink) and stationary (green) growth phases. Asterisks indicate statistically significant differences (*P* < 0.05).

Disturbance of NO homeostasis was accompanied by coordinated changes in intracellular redox systems. Several redox-associated proteins were differentially abundant in the Δ*tspO* mutant. NAD kinase (NADK) was more abundant during both the E and S phases (Fig. 7A; Table S2). Increased abundance of bacilliredoxin (Q81E36), a thiol-disulfide oxidoreductase involved in protein repair (21), was also observed, along with altered abundance of enzymes involved in thiol metabolism, including cysteine synthase A (Q818A2) and cysteine synthase B (Q81F39). The persistent decrease of cysteine synthase A may compromise bacillithiol production (22), potentially increasing cellular redox vulnerability. Increased abundance of aldehyde dehydrogenase (ALDH; Q81AK8), nitronate monooxygenase (Q813U3), and the FMN-dependent NADH-quinone oxidoreductase (AZOR2; Q81E02) further highlights enrichment of proteins associated with detoxification and redox-related functions (23–26). In line with these proteomic alterations, Δ*tspO* cells displayed a higher NADP^+^/NAD^+^ ratio during exponential growth (Table 1), while NADPH levels were markedly reduced, suggesting increased consumption of reducing power under stress conditions. Together, these findings indicate coordinated alterations in NO homeostasis and redox-associated pathways in the Δ*tspO* mutant during anaerobic fermentation.

### BcTSPO modulates phosphate balance and metal ion homeostasis

Several proteomic markers of phosphate limitation were strongly increased in the Δ*tspO* mutant, particularly at the E phase. These included a phosphate-binding protein, the Pit accessory protein, polyphosphate kinase (PPK1), and the phosphate starvation-inducible protein PhoH (Fig. 7B). This response may reflect increased phosphate demand under redox stress. In parallel, the Δ*tspO* mutant showed broad alterations in metal ion homeostasis (Fig. 7C). Ion transporters involved in calcium, potassium, and divalent cation uptake—including a Ca²^+^-transporting ATPase (27), the potassium uptake protein KtrA (28), and the Mg²^+^/Co²^+^/Ni²^+^ transporter CorA (29)—were more abundant at the E phase. The manganese-responsive regulator MntR was also increased, suggesting perturbations in Mn homeostasis (30). In contrast, proteins associated with iron homeostasis were less abundant. Notably, ferritin, the major iron storage protein (31) showed decreased abundance, as did the Fur regulator (Fig. 7C). These changes likely reduce intracellular iron storage capacity, potentially worsening redox imbalance and contributing to nitrosative stress. Together, these observations indicate altered phosphate and metal ion homeostasis in the Δ*tspO* mutant during anaerobic fermentation.

### BcTSPO modulates the exoproteome and surface-associated protein landscape

**Fig 8:**
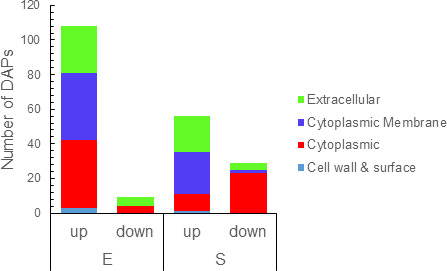
Subcellular distribution of differentially abundant proteins detected in the exoproteome of the Δ*tspO* mutant. Bar plots show the number of differentially abundant proteins (DAPs) detected in the exoproteome during exponential (E) and stationary (S) growth phases, categorized according to their predicted subcellular localization. Subcellular localization was predicted using DeepLocPro 1.0. Note that “extracellular” includes both secreted and surface-associated proteins, as defined by the prediction algorithm.

To determine whether intracellular proteomic remodeling extended to the exoproteome and surface-associated proteins, we analyzed the predicted subcellular localization of the differentially abundant proteins (DAPs). Most DAPs were predicted to be cytoplasmic or associated with the cytoplasmic membrane, while a smaller fraction was classified as extracellular or cell wall-associated according to DeepLocPro (Fig. 8). This distribution indicates that the absence of BcTSPO affects proteins from multiple cellular compartments, not only those actively secreted. We next focused on the 33 proteins consistently altered across both E and S phases, which likely constitute a stable exoproteomic signature of the Δ*tspO* mutant (Table 2). This set included 10 cytoplasmic, 12 membrane-associated, one cell wall-associated, and 10 predicted extracellular proteins. Several corresponded to genuine secreted or surface-exposed factors, such as the flagellar hook proteins FlgK and FlgL, whereas others (e.g., MutT, FtsK) were likely cytoplasmic or membrane-associated despite their extracellular prediction. Among the proteins persistently more abundant in the Δ*tspO* mutant, four were involved in iron acquisition, including two ferrichrome-binding and one iron (III)-dicitrate-binding proteins, as well as a cytoplasmic CDGSH-domain iron-binding protein. Their coordinated overproduction suggests a compensatory response to intracellular iron limitation, possibly linked to impaired heme metabolism. Proteolytic and surface-active enzymes–including bacillolysin, DegS, a neutral metalloprotease, and sphingomyelinase C—were also more abundant in the Δ*tspO* mutant, consistent with enrichment of extracellular or surface-associated enzymatic functions. The only enterotoxin consistently increased was the L2 component of the hemolysin BL complex, suggesting selective modulation of virulence factor release. Together, these findings indicate alterations in extracellular protein composition and surface-associated functions in the Δ*tspO* mutant during anaerobic fermentation.

**TABLE 2:** Proteins consistently affected in the exoproteome of the Δ*tspO* mutant

Change in Δ*tspO*^*a*^	Gene name	UniProt ID	Name	GO biological process	COG category^*b*^	Functional description
Cell wall and surface associated proteins						
↑	BC_2506	Q81D73	–	Homophilic cell adhesion	E	Bacillolysin
Cytoplasmic proteins						
↓	BC_3977	Q819J9	RnjA	mRNA and rRNA processing	S	Ribonuclease J
↑	BC_4163	Q818T0	Ptb	Butyrate metabolic process	C	Phosphate butyryltransferase
↓	BC_3972	Q819K4	PdhB	–	C	Pyruvate dehydrogenase E1 component β subunit
↑	BC_4313	Q818E8	GrpE	Protein folding	O	HSP-70 cofactor
↑	BC_4521	Q817L8	TrxA	Cell redox homeostasis	O	Thioredoxin
↑	BC_0149	Q81J24	RpmD	Translation	J	50S ribosomal protein L30
↑	BC_4991	Q815Y3	GcvH	Glycine decarboxylation	E	Glycine cleavage system H protein
↑	BC_0042	Q81JA9	AbrB	Transcriptional regulation	K	Transcription state regulatory protein
↑	BC_4049	Q819D6	PtsH	Sugar phosphotransferase system	G	Phosphocarrier protein HPr
↑	BC_4108	Q818X9	–		S	Iron-binding zinc finger CDGSH type domain-containing protein
Membrane-associated proteins						
↑	BC_1179	Q81GL6	OppA	Peptide and protein transport	E	Periplasmic oligopeptide-binding protein
↑	BC_3916	Q812W3	PbpB	Peptidoglycan biosynthetic process	M	Serine-type D-Ala-D-Ala carboxypeptidase
↑	BC_3600	Q81AG8	DegS	Proteolysis	O	Serine endoprotease
↑	BC_0215	Q81IX9	OppA	Peptide and protein transport	E	Periplasmic oligopeptide-binding protein
↑	BC_3791	Q81A05	TcsA		S	Nucleoside-binding protein
↑	BC_1043	Q81GY5	PrsA	Protein folding	M	Foldase protein
↑	BC_3070	Q81BS6	LepB	Signal peptide processing	U	Signal peptidase I
↑	BC_3738	Q81A53	FeuA	Iron coordination entity transport	P	Iron(III) dicitrate-binding protein
↑	BC_5200	Q815F9	–	Cell wall organization	K	Transcriptional regulator, LytR family
↑	BC_0216	Q81IX8	OppA	Peptide and protein transport	E	oligopeptide-binding protein
↑	BC_4363	Q818A5	FhuD	Iron coordination entity transport	P	Ferrichrome-binding protein
↑	BC_5380	Q814P4	–	Iron coordination entity transport	P	Ferrichrome-binding protein
Extracellular proteins						
↑	BC_3104	Q81BP7	HblL2		S	Hemolysin BL component L2
↑	BC_2735	Q81CL9	–	Proteolysis	E	Neutral metalloproteinase
↑	BC_1636	Q81FF4	FlgK	Bacterial-type flagellum assembly	N	Flagellar hook-associated protein 1
↓	BC_0896	Q813X9	–		G	S-layer protein/peptidoglycan endo-beta-N-acetylglucosaminidase
↑	BC_0671	Q81HW0		Killing of cells of another organism	S	Sphingomyelinase C
↑	BC_1637	Q81FF3	FlgL	Bacterial-type flagellum assembly	N	Flagellar hook-associated protein 3
↓	BC_0902	Q81HB4	LytC1	Peptidoglycan catabolic process	M	S-layer protein/N-acetylmuramoyl-L-alanine amidase
↑	BC_3527	Q81AN5	MutT			DNA mismatch repair protein
↑	BC_2682	Q81CR5	–	Polysaccharide catabolic process	G	Glucanase
↑	BC_2186	Q81E10	–		S	Cell division protein

^*a*^ Arrows indicate direction of change in protein abundance relative to WT (↑ increase; ↓decrease). Values are provided in Table S1.

^*b*^ COG categories: C, energy production and conversion; E, amino acid transport and metabolism; F, nucleotide transport and metabolism; G, carbohydrate transport and metabolism; H, coenzyme transport and metabolism; I, lipid transport and metabolism; J, translation, ribosomal structure and biogenesis; K, transcription; L, replication, recombination and repair; M, cell wall/membrane/envelope biogenesis; O, post-translational modification, protein turnover and chaperones; P, inorganic ion transport and metabolism; Q, secondary metabolite biosynthesis, transport and catabolism; T, signal transduction mechanisms; U, intracellular trafficking and secretion; V, defense mechanisms. “–” indicates that the information is not available.

## DISCUSSION

While TSPO has been extensively studied under oxic conditions, its physiological role during anoxia remains largely unexplored. Here, we show that the absence of BcTSPO disrupts porphyrin and iron homeostasis and is associated with intracellular redox imbalance under fermentative anoxic conditions. These disturbances are accompanied by characteristic nitrosative stress features, extensive proteome remodeling, impaired fermentative growth, and signs of membrane perturbation.

The MOD medium used in this study is not supplemented with metal ions, leading to mild iron limitation (32). Under such conditions, synthesis of heme and siroheme is particularly sensitive, as iron insertion occurs at the final biosynthetic steps (Fig. 5). In this context, the ∆*tspo* mutant accumulated higher levels of porphyrin intermediates than the WT during exponential growth. Concomitantly, the mutant displayed reduced abundance of the iron-responsive regulator Fur and increased abundance of exoproteins involved in iron acquisition, a pattern consistent with functional iron starvation. These observations indicate impaired coordination between porphyrin metabolism and intracellular iron utilization in the absence of BcTSPO. TSPO has previously been proposed to contribute to porphyrin degradation (15, 16, 33), possibly through ROS-dependent mechanisms (34). However, such activity is unlikely under our low-ROS, fermentative anoxic conditions. Rather, our data suggest a role for BcTSPO in maintaining proper functioning of the coproporphyrin-dependent branch of heme biosynthesis, thereby limiting the accumulation of upstream intermediates such as coproporphyrin and uroporphyrin. One possible explanation is that BcTSPO facilitates coordination of enzymatic steps within this pathway, according to recent evidence that multiprotein assemblies promote intermediate channeling and iron delivery during heme formation in gram-positive bacteria (35).

In this context of impaired heme biosynthesis and functional iron limitation, alterations in nitrogen metabolism become particularly significant. Although no exogenous nitrate or nitrite was provided and canonical bNOS expression was repressed, the ∆*tspo* mutant exhibits features consistent with a nitrosative stress signature. This is supported by the increased abundance of Hcp, a heme-independent NO detoxifier (18, 36), and by the increased abundance of nitrate (NarGH) and nitrite (NasDE) reductase, which require heme or siroheme cofactors. Their increased abundance likely reflects transcriptional responses to NO accumulation, mediated by redox-sensitive regulators such as Fnr and Fur (20, 37–40). The lack of induction of siroheme-dependent sulfite reductase further supports the idea that this is a targeted response to nitrosative stress rather than a general increase in siroheme demand (20). The simultaneous repression of bNOS and upregulation of arginase (Fig. 6) may restrict intracellular NO precursor availability. However, low-level endogenous NO production via alternative routes (41, 42) may become damaging in a context of disrupted redox buffering.

Multiple proteomic changes further support redox imbalance in the Δ*tspo* mutant. The increased abundance of NAD kinase, together with the altered NADP^+^/NAD^+^ ratio and reduced NADPH levels measured in the Δ*tspO* strain, is consistent with a shift in intracellular redox cofactor balance, a hallmark of redox adaptation. Along with reduced translation machinery, this suggests a reallocation of cellular resources toward stress mitigation. Changes in carbon flux and increased acetate production reinforce the idea of a metabolic rewiring to maintain redox balance. The increased abundance of aldehyde dehydrogenase (ALDH) is consistent with enhanced detoxification of lipid-derived aldehydes (43), while induction of a bacilliredoxin-like protein supports activation of thiol-based redox defense systems, as previously observed in oxidative stress settings (44).

Broader disruptions in ion homeostasis were also evident. Phosphate transport systems were induced even in the absence of extracellular phosphate depletion, possibly reflecting increased ATP turnover associated with antioxidant responses (45). Proteins involved in manganese, potassium, calcium, and magnesium transport were altered, highlighting adjustments in metal homeostasis that accompany redox pressure. The increased abundance of a calcium-transporting ATPase is particularly notable. Although calcium signaling is less characterized in bacteria than in eukaryotes, Ca²^+^ fluxes have been implicated in bacterial stress responses and membrane perturbation (27). TSPO has been linked to mitochondrial Ca²^+^ homeostasis in eukaryotes (46). Therefore, increased Ca²^+^-ATPase expression in the Δ*tspo* mutant may reflect a compensatory mechanism to counterbalance membrane or ionic imbalances, suggesting a potentially conserved functional theme.

The effects of *tspO* deletion also differed between growth phases. During exponential growth, the Δ*tspo* mutant displayed pronounced redox and metabolic pressure, consistent with higher *tspO* expression and increased vulnerability during active metabolism. In the stationary phase, envelope stress became more prominent, reflected in the induction of cell wall-modifying enzymes and surface-associated proteins, likely representing a response to cumulative redox and nitrosative damage.

In conclusion, our findings support a role for BcTSPO in coordinating porphyrin and iron homeostasis under fermentative anoxic conditions. By contributing to the proper functioning of the heme biosynthetic pathway and limiting the accumulation of porphyrin intermediates, BcTSPO links porphyrin metabolism to intracellular iron utilization. Its absence is associated with impaired coordination of these processes, coinciding with features of functional iron limitation, redox imbalance, and secondary metabolic stress that collectively compromise cellular fitness. This role—centered on the integration of tetrapyrrole and metal metabolism rather than direct detoxification—may reflect an ancestral function of TSPO. Such a mechanism may have been conserved and later adapted in eukaryotic organelles, where TSPO contributes to redox regulation and stress signaling. Together, these findings support the view that TSPO functions as a metabolic integrator linking iron, porphyrin, and redox homeostasis across domains of life.

## MATERIALS AND METHODS

### Bacterial strains, media, and growth conditions

The *B. cereus* wild-type strain ATCC 14579, the isogenic ∆*tspO* mutant, and the ∆*tspO* strain harboring the *tspO* expression plasmid (14), together with the corresponding vector control, were grown in MOD medium supplemented with 30 mM glucose (MODG) as the carbon source (32). Anaerobic batch cultures were carried out at 37 °C in a 2 L bioreactor (Applikon) containing 1.5 L of MODG medium. The pH was maintained at 7.2 through the automatic addition of 3 M KOH and 1 M HCl, with continuous agitation at 200 rpm. Strict anaerobic conditions (pO_₂_ = 0%) were achieved by continuously sparging the medium with pure nitrogen gas (10 mL/h) pre-treated through a Hungate column (47). The inoculum consisted of 150 mL of an exponentially growing anaerobic culture, harvested by centrifugation, washed twice, and resuspended in fresh MODG medium to reach an initial optical density at 600 nm (OD₆₀₀) of 0.02. Three biological replicates were performed for each strain.

### Growth conditions and sample preparation

Bacterial growth was monitored spectrophotometrically at 600 nm (OD₆₀₀). The maximal specific growth rate (µ_max_) was calculated using the modified Gompertz equation (48). Culture samples (50 mL) were collected at the exponential phase (OD₆₀₀ = 0.23 ± 0.03 for WT and 0.22 ± 0.00 for Δ*tspO*) and at the stationary phase (OD₆₀₀ = 1.79 ± 0.01 for WT and 1.23 ± 0.02 for Δ*tspO*). Cells and culture supernatants were separated by centrifugation (10,000 × *g*, 10 min, 4°C). Cell pellets were resuspended in phosphate-buffered saline (PBS) and stored at –20°C. Supernatants were filtered as previously described (17, 49). Cellular and extracellular proteins were extracted from cell pellets and filtered supernatants, respectively, according to previously described protocols (14, 17).

### Metabolite analysis

The concentrations of glucose, lactate, ethanol, formate, and acetate were determined in filtered culture supernatants using Enzytec Fluid kits (R-Biopharm, Saint-Didier-au-Mont-d’Or, France). Succinate was quantified using the Megazyme assay kit (Megazyme, Ireland).

Intracellular concentrations of NAD(P)^+^ and NAD(P)H were measured using the EnzyChrom assay kits (BioAssay Systems, Hayward, USA) following the manufacturer’s instructions. Cells (~10⁵) in exponential growth phase were harvested by centrifugation (10,000 × *g*, 5 min, 4°C) and washed twice with cold PBS (pH 7.5). Cell pellets were resuspended either in acidic or basic extraction buffers provided in the kit to extract oxidized or reduced pyridine nucleotides, respectively. NAD(P)^+^ and NAD(P)H concentrations were determined via a lactate dehydrogenase (LDH)-based cycling reaction, in which NAD(P)H reduces a formazan (MTT) dye, allowing spectrophotometric quantification.

Porphyrin concentrations and intracellular reactive oxygen species (ROS) levels were measured as previously described (14).

### Proteomic analysis of cellular and extracellular proteins

Proteins from cell pellets and culture supernatants were extracted and digested with trypsin as described previously (14, 17). Peptide samples were analyzed using an Ultimate 3000 nanoLC system coupled to a Q-Exactive HF mass spectrometer (Thermo Scientific). For both cellular and extracellular fractions, peptides (10 µL) were first trapped on an Acclaim PepMap 100 C18 precolumn and separated on a nanoscale C18 analytical column (75 µm × 500 mm, 3 µm, 100 Å) using a binary solvent system (buffer A: 0.1% formic acid in water; buffer B: 0.1% formic acid in 80% acetonitrile). Elution gradients were adjusted depending on the sample type: 90 min (4%–25% B in 75 min, 25%–40% B in 15 min) for cellular proteins, and 60 min (4%–25% B in 50 min, 25%–40% B in 10 min) for secreted proteins. Data were acquired in data-dependent acquisition mode using a Top20 method with a dynamic exclusion of 10 s. MS scans were recorded in the Orbitrap at a resolution of 60,000 over an *m*/*z* range of 350–1,500. The 20 most intense precursor ions (charge states 2+ and 3+) were selected for HCD fragmentation and MS/MS acquisition (resolution 15,000). MS/MS spectra were searched against the *B. cereus* ATCC 14579 UniProt database using Mascot (v2.5.1, Matrix Science), with a precursor mass tolerance of 5 ppm and fragment mass tolerance of 0.02 Da. Carbamidomethylation of cysteine was set as a fixed modification, and methionine oxidation as well as asparagine/glutamine deamidation were included as variable modifications. Protein identifications were validated with a minimum of two peptides per protein, at least one unambiguous peptide, and a false discovery rate below 1%. Relative protein abundances were compared between wild-type and ∆*tspO* strains using the TFold statistical test (14), considering significant differences at adjusted *P* < 0.05 and |log_2_ fold change| > 0.58.

### Gene expression analysis by RT-qPCR

Total RNA was extracted from *B. cereus* cells using the Monarch Total RNA Miniprep Kit (New England Biolabs) according to the manufacturer’s instructions. RNA concentration and purity were assessed using a NanoDrop spectrophotometer. Quantitative reverse transcription PCR (RT-qPCR) was carried out using the Takyon Ultra One-Step RT-PCR Kit with SYBR Green (Eurogentec, France), following the manufacturer’s protocol. Reactions were performed with 10 ng of total RNA as template, and 200 nM forward and reverse primers in a 96-well plate using a Mastercycler ep realplex thermal cycler (Eppendorf, Hamburg, Germany). Gene-specific primers are listed in Table S3. Negative controls (no-template and no-reverse-transcriptase) were included to monitor for DNA contamination and non-specific amplification. All reactions were run in technical duplicates. The gene *ssu* was used as endogenous control (Table S3). Fold changes in expression were expressed as 2⁻^ΔΔCt^ values. Data are presented as mean ± SD from three independent biological replicates with two technical replicates each. Statistical significance of expression differences between conditions was assessed using Student’s *t*-test, with a *P*-value <0.05 considered statistically significant.

## Data Availability

The proteomics data have been deposited in the ProteomeXchange Consortium via the PRIDE repository under the accession numbers PXD055392 and PXD055393.

## SUPPLEMENTAL MATERIAL

Fig. S1 msystems.01738-25-s0001.tif Validation of Δ*tspO* phenotypes and complementation under fermentative anoxic conditions.

Table S1 msystems.01738-25-s0002.xlsx Proteins identified in the cellular proteomes of WT and Δ*tspO* cells.

Table S2 msystems.01738-25-s0003.xlsx Proteins identified in the exoproteome of WT and Δ*tspO* strains.

Table S3 msystems.01738-25-s0004.docx Primers used for quantitative real-time PCR.

ASM does not own the copyrights to Supplemental Material that may be linked to, or accessed through, an article. The authors have granted ASM a non-exclusive, world-wide license to publish the Supplemental Material files. Please contact the corresponding author directly for reuse.
