# Supplementary figures and images for: TSPO safeguards porphyrin–iron balance under anoxic conditions in Bacillus cereus
Source: mSystems. 2026 Apr 13;11(5):e01738-25. doi: 10.1128/msystems.01738-25 (PMC13185565; doi:10.1128/msystems.01738-25)

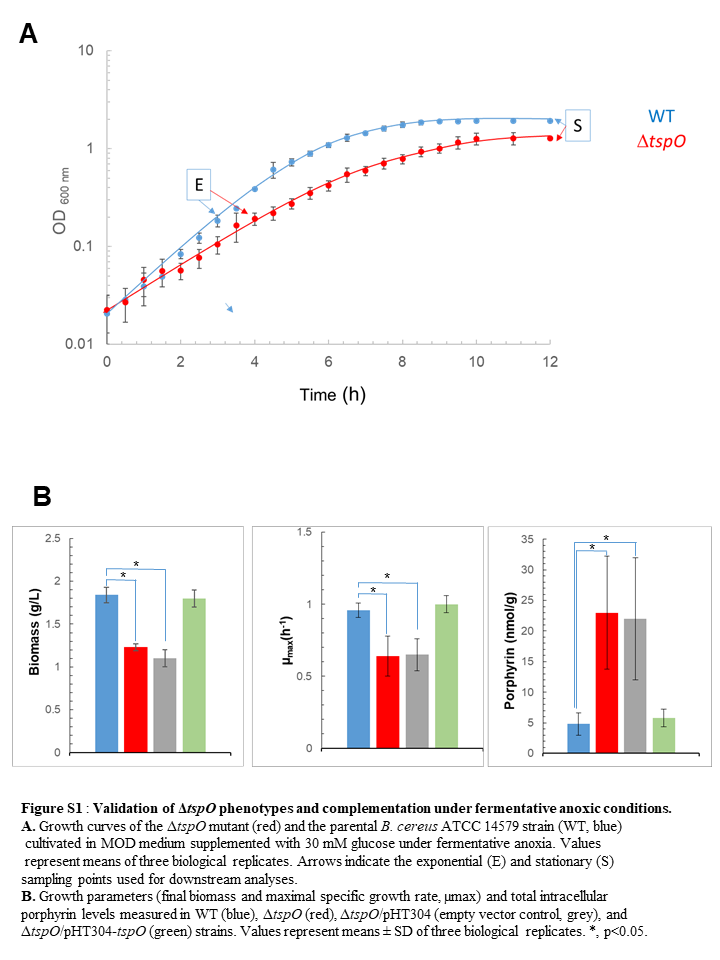

Supplement: Fig. S1 — Validation of ΔtspO phenotypes and complementation under fermentative anoxic conditions. [file msystems.01738-25-s0001.tif]
