# Supplementary material for: TSPO safeguards porphyrin–iron balance under anoxic conditions in Bacillus cereus
Source: mSystems. 2026 Apr 13;11(5):e01738-25. doi: 10.1128/msystems.01738-25 (PMC13185565; doi:10.1128/msystems.01738-25)
Supplement: Table S3 — Primers used for quantitative real-time PCR. [file msystems.01738-25-s0004.docx]

Table S3: Primers used for quantitative real-time PCR

| Gene | Forward primer 5’🡪3’ | Reverse primer 5’🡪3’ |
| --- | --- | --- |
| *tspO* | AAAAGCCGTCTTGGACACCT | TGGTATTAGCAACCATGCCGA |
| *gntR* | CAAAACGTGCCCACTCAGAA | GTAGTGCAAGTTCGCCCATT |
| *cydA* | GGTCGTGCGCAAATGAATGA | GCGCCGTTGTAATTGTTCCA |
| *fnr* | GCAAACGAAGTTCCGAGATT | GCAGAGCAATCTTCACAAGC |
| *ssu* | GCAAGGCTGAAACTCAAAGG | TGTAGCCCAGGTCATAAGGG |
